# A Community-Engaged Approach to Developing an mHealth HIV/STI and Drug Abuse Preventive Intervention for Primary Care: A Qualitative Study

**DOI:** 10.2196/mhealth.4620

**Published:** 2015-12-18

**Authors:** David Cordova, Jose A Bauermeister, Kathryn Fessler, Jorge Delva, Annabelle Nelson, Rachel Nurenberg, Frania Mendoza Lua, Francheska Alers-Rojas, Christopher P Salas-Wright, Youth Leadership Council

**Affiliations:** ^1^ School of Social Work University of Michigan Ann Arbor, MI United States; ^2^ School of Public Health University of Michigan Ann Arbor, MI United States; ^3^ The Corner Health Center Ypsilanti, MI United States; ^4^ School of Human & Organizational Development Fielding Graduate University Santa Barbara, CA United States; ^5^ Department of Psychology University of Michigan Ann Arbor, MI United States; ^6^ School of Social Work The University of Texas at Austin Austin, TX United States

**Keywords:** adolescent, primary prevention, HIV, STI, mHealth, telemedicine, primary health care, drug users, sexually transmitted infections

## Abstract

**Background:**

Despite ongoing prevention efforts, HIV and other sexually transmitted infections (HIV/STIs) and drug use remain public health concerns. Urban adolescents, many of whom are underserved and racial minorities, are disproportionately affected. Recent changes in policy, including the Affordable Care Act, and advances in technology provide HIV/STI and drug abuse prevention scientists with unique opportunities to deliver mobile health (mHealth) preventive interventions in primary care.

**Objectives:**

The purpose of this community-engaged study was to develop an mHealth version of the Storytelling for Empowerment preventive intervention for primary care (hereinafter referred to as “S4E”).

**Methods:**

A total of 29 adolescents were recruited from a youth-centered primary care clinic in Southeast, Michigan, to participate in qualitative interviews. Participants were predominantly African American (n=19, 65.5%) and female (n=21, 72.4%) with a mean age of 16.23 (SD 2.09). The principles of community-based participatory research (CBPR), in conjunction with agile software development and the recommended core prevention principles of the National Institute on Drug Abuse (NIDA) were employed during S4E development. CBPR principles are aimed at improving the effectiveness of research by addressing locally relevant health problems, working with community strengths, and translating basic science into applied research. Complementing this approach, the NIDA prevention principles are derived from decades of drug abuse prevention research aimed at increasing the effectiveness and uptake of programs, through the development of culturally specific interventions and ensuring the structure, content, and delivery of the intervention fit the needs of the community. Data were analyzed using thematic analysis.

**Results:**

A total of 5 themes emerged from the data: (1) acceptability of the mHealth app to adolescents in primary care, (2) inclusion of a risk assessment to improve clinician-adolescent HIV/STI and drug use communication, (3) incorporation of culturally specific HIV/STI and drug use content, (4) incorporation of interactive aspects in the app to engage youth, and (5) perspectives on the appearance of the app.

**Conclusions:**

There is a dearth of mHealth HIV/STI and drug abuse preventive interventions for primary care. Incorporating the principles of CBPR in conjunction with agile software development and NIDA-recommended core prevention principles may be helpful in developing culturally specific mHealth interventions. An important next step in this program of research is to examine the feasibility, acceptability, and efficacy of S4E on adolescent sexual risk and drug use behaviors, and HIV/STI testing. Implications for prevention research and primary care practice are discussed in the context of the Affordable Care Act and technological advances.

## Introduction

Despite ongoing prevention efforts, HIV/STI and drug use remain public health concerns in the United States. Compared with older age groups, for whom incidence has remained stable, HIV/STI incidence among youth (aged 15-19) continues to increase [1,2]. The disproportionate incidence of HIV/STI during adolescence may be attributed to increased risk taking during this developmental period [3], including the onset of sexual and drug use behaviors [4,5]. Disparities among adolescents also suggest that underserved racial/ethnic minorities, many who live within urban contexts, are more likely to engage in HIV/STI risk behaviors, including drug use [4,5], and use of alcohol and drugs prior to sexual intercourse [4]. These drug use patterns may increase youth’s engagement in sexual risk behaviors (eg, inconsistent condom use) and subsequent exposure to HIV/STI infections. Given the dearth of mHealth interventions that assess and target multiple HIV/STI adolescent risk behaviors (ie, drug use and sexual risk behaviors) and increase HIV/STI testing [6-8], the purpose of this study was to develop an mHealth version of the Storytelling for Empowerment preventive intervention.

A face-to-face intervention, Storytelling for Empowerment (SFE) is registered as a best practice with the Substance Abuse and Mental Health Services Administration (SAMHSA) National Registry of Evidence-Based Programs and Practices. SFE was developed in 1995 with a grant from the SAMHSA Center for Substance Abuse Prevention High Risk Youth Program and was further evaluated through three subsequent SAMHSA grants. SFE aims to increase HIV/STI and drug use self-efficacy, including condom use skills, knowledge, HIV/STI testing, and communication. SFE has been shown to prevent and reduce HIV/STI risks, including drug use and sexual risks [9-11]. SFE consists of 6 core sections, including knowledge power (eg, effects of drug use), skill power (eg, decision making), personal power (eg, self-efficacy), character power (eg, empowerment), cultural power (eg, community protective factors), and future power (eg, clinician-adolescent HIV/STI communication). Although the original SFE intervention focused on licit and illicit drug use outcomes [9,10], additional sections on sexual risk behaviors have been developed and evaluated [11].

The ecodevelopmental framework [12-14] postulates that adolescents are embedded in integrated ecological systems and may experience heightened vulnerability to negative health outcomes if they lack developmentally appropriate skills required to navigate these different systems. Building on developmental and social interaction theories, ecodevelopmental theory further posits that these systems influence and are influenced by the adolescent over time [12]. Within this framework, researchers have proposed empowerment strategies that link youth’s strengths and proactive behaviors to natural helping systems, while offsetting risk behaviors [15]. While most ecodevelopmental interventions to date have focused on the family microsystem (eg, improving family functioning and parent-adolescent communication) [14,16,17], fewer youth-driven interventions have been tested within the primary care microsystem. Thus, for the purposes of this study, we focus on the primary care microsystem. Recommendations issued by the United States Preventive Services Task Force have highlighted the need for primary care clinicians to provide screening for HIV/STI risk behaviors (eg, drug use) and HIV/STI status [18]; however, few HIV/STI and drug abuse preventive interventions for primary care exist [19,20].

Recent shifts in public health policies, including the Affordable Care Act (ACA), combined with advances in technology, provide prevention scientists and practitioners alike with innovative opportunities to implement HIV/STI and drug abuse preventive interventions. For example, mobile health (mHealth) interventions, including apps and primary care settings, offer novel approaches and contexts to deliver prevention programs [6,7,21]. Yet, few researchers have developed mHealth HIV/STI and drug abuse preventive interventions for primary care that focus on curbing risk practices (eg, drug use, condomless sex) and increasing HIV/STI testing [7,8,19]. Even fewer researchers have employed the principles of community-based participatory research (CBPR) to inform the development and adaptation of efficacious HIV/STI and drug abuse preventive interventions into an mHealth platform for primary care. A community-engaged approach may aid in the development of theory-driven, culturally specific mHealth interventions for the targeted community [22]. The purpose of this community-engaged study was to adapt face-to-face SFE [9-11], an efficacious adolescent HIV/STI and drug abuse preventive intervention, into an mHealth modality for primary care (S4E). For the purposes of this study, S4E was culturally specific such that the development was youth driven, targeted a specific health care center, and addressed the local HIV/STI and drug use community needs.

## Methods

This study was guided by the principles of CBPR (eg, build on the strengths and resources of the community [23]). As part of this approach, the Youth Leadership Council (YLC) was engaged in all aspects of the study, including proposal submission, development of the interview guide, participant recruitment, identification of content for app development, and manuscript preparation. The YLC is a diverse, youth-led group that aims to research community health issues, use media and the arts to advocate for change, and partner with universities to make the primary care clinic and surrounding community a healthier place for adolescents.

### Sampling and Recruitment

Initially, we aimed to recruit younger adolescents (ie, 13-15 years of age). However, at the recommendation of the YLC, we changed our approach and targeted a study sample that was more representative of the primary care clinic population (ie, 12-21 years of age). Therefore, to be included in this study, participants had to (1) be between 13 and 18 years of age, and (2) provide assent and parental consent (if under 18 years of age). Potential participants were recruited from the waiting room of the primary care clinic via strategies found to be effective when conducting qualitative research with vulnerable populations [24]. As such, recruitment procedures included establishing relationships with the primary care clinic, face-to-face flier distribution, word of mouth, and snowballing approaches. For example, participants reached out to their peers who also received services at the clinic and informed them about the study. Data collection occurred between December 2013 and October 2014, and was approved by our University’s Institutional Review Board.

A total of 29 adolescents participated in this study. Participants were primarily African American (n=19, 65.5%), followed by non-Hispanic white (n=7, 24.1%), Hispanic (n=2, 6.8%), and other (n=1, 3.4%). The majority of adolescents were female (n=21, 72.4%) with a mean age of 16.21 (SD 2.12).

### Interview Guide

The interview guide consisted of 3 open-ended grand tour questions aimed at minimizing questioning bias and privileging participants’ voices: “Would you please describe your thoughts about participating in an intervention delivered on a mobile device through your primary care clinic that is designed to prevent HIV/STI and drug abuse in adolescents?” “What content would you find most helpful to include and engage people your age?” “Would you please describe your thoughts regarding the design aspects and what the app should look like?” The grand tour questions were followed by probes to gather in-depth data (eg, “How long should the app last?”). Furthermore, we included probes to ensure that our questions were tethered to the SFE modules and to gather in-depth data on how they could be translated to an app-like experience. For example, our grand tour question, “What content would you find most helpful to include and engage people your age?” was followed by probes such as, “What information with respect to HIV/STI and drug use would you find helpful in an app?” Data gathered from this probe were then applied to the development of the knowledge aspects of the app, which correspond to knowledge-, skill-, personal-, character-, cultural-, and future-power aspects of SFE. To ensure that we did not overlook any design, content, or process aspects that adolescents believed to be important in the development of the app, participants were asked at the conclusion of the interview to discuss any relevant topics that were not addressed. Consistent with the iterative process, the interview guide was gradually modified after each interview to include probes that participants identified as relevant and that were not initially included in the interview guide (eg, “What are your thoughts on including a risk assessment to promote clinician-adolescent communication?”).

### Procedures

Focus group and individual interviews were utilized for data collection. Focus group interviews have been shown to be empowering for vulnerable populations, as the group processes allow for a shared experience among participants and may produce data that cannot be obtained solely through individual interviews [24,25]. A total of 9 focus group interviews (n=25) were conducted and ranged in size from 2 to 6 participants. A limitation to focus group interviews, however, is that not all adolescents may feel comfortable discussing topics related to sexual risk and drug use behaviors in a group setting. Therefore, we also conducted individual interviews (n=4). All interviews were conducted in a private room located in the primary care clinic. Each interview was digitally recorded, lasted between 45 and 75 minutes, and was facilitated by the lead author and trained research assistants. Prior to each interview, a meal was shared between the research team and participants, aimed at facilitating informal conversations and building rapport [24]. Participants were provided bus tokens and received a US $20 incentive.

Guided by the principles of agile software development [26], the qualitative interviews and app programming were synergistic. Agile software development aims for continuous design improvement and testing based on rapid feedback and change [26]. The development of the HIV/STI and alcohol/drug modules was informed by the qualitative interviews in conjunction with relevant National Institute on Drug Abuse (NIDA) core prevention principles. For the purposes of this study, we employed NIDA-recommended prevention principles 1, 2, 3, 4, 8, 9, 12, 13, 15, and 16 [27] (Table 1). While we conducted interviews to identify HIV/STI and drug use-specific content to include in the app, the interface was being built. Thus, the backend system and framework (eg, code, database, design) were developed as qualitative data were collected. As part of the iterative process, weekly meetings were held with the programmer to discuss all aspects of the app development, including content, framework, and UX (user experience). Once the qualitative interviews were completed and content was finalized, the messaging was incorporated into the interface.

**Table 1:** Incorporating the NIDA prevention principles into the app development.

NIDA prevention principles	How this was accomplished
Principle 1: Programs should enhance protective factors and prevent and reduce risk factors.	S4E aims to improve clinician-adolescent HIV/STI and drug use communication, condom use and drug use resistance self-efficacy, and increase HIV/STI testing.
Principle 2: All forms of licit and illicit drug abuse should be addressed.	The Alcohol/Drugs module includes both licit and illicit drugs, including abuse of medication without a doctor’s prescription.
Principle 3: Programs should address culturally specific risk and protective factors and licit and illicit drug use in the targeted community.	In-depth qualitative data informed the development of culturally specific content, including risk and protective factors and licit and illicit drug use.
Principle 4: Prevention programs should be tailored to address risks specific to the targeted community, including age, gender, and race, to improve program effectiveness.	The community-engaged approach aimed to identify risks specific to the targeted community.
Principle 8: Programs targeting high-school students should increase social competence skills, including communication, self-efficacy, and drug-resistance skills.	S4E aims to improve clinician-adolescent HIV/STI and drug use communication, self-efficacy, and drug-resistance skills.
Principle 9: Programs aimed at key transition points, including the transition to young adulthood, may yield beneficial effects.	S4E targets those in adolescence and young adulthood, a transitional period marked by increased risk taking.
Principle 12: Adapted programs should retain the core elements of the original research-based intervention.	We retained the core elements (knowledge development, self-efficacy, and communication) of the face-to-face Storytelling for Empowerment intervention.
Principle 13: Programs should be long-term, including the use of booster sessions.	An mHealth app may provide opportunities for adolescents to engage in long-term prevention, including booster sessions.
Principle 15: Prevention programs should include interactive exercises to work toward optimally effective interventions.	S4E incorporates interactive exercises. Additionally, we plan to develop a clinician component to allow interaction and retrieval of adolescents’ risk assessment data, with an aim of improving clinician-adolescent interaction, including HIV/STI and drug use communication.
Principle 16: Research-based programs can be cost effective.	mHealth apps, including S4E, have the potential to be cost effective through greater reach to populations disproportionately affected by HIV/STI and drug use, as well as by relieving some of the responsibilities and sparing resources in a clinic setting.

### Analytic Approach

Trained research assistants transcribed verbatim the digital audio recordings of each interview. A different research team member then reviewed these transcripts for accuracy. Data were transferred to NVivo 10 (QSR International Pty Ltd) for storage, organization, and analysis. Data analysis followed the tenets of thematic analysis, which consisted of 6 sequential steps [28,29]. First, the research team familiarized themselves with the data by transcribing all interviews and reviewing each transcription prior to the next scheduled interview. Second, the research team met weekly and generated an initial list of codes to discuss. Third, the initial codes were collated into emerging themes. Fourth, the team generated a thematic table of the analyses and checked the extent to which the emerging themes reflected the coded extracts and data. Fifth, the team generated clear definitions and names for each theme and overall analysis. Lastly, the team identified the most compelling examples relating back to the analysis of the study [28,29].

The analytic process yielded 252 initial codes. Of these, 5 themes were identified (see Table 2 for the analytic process): (1) an mHealth app is acceptable to adolescents in primary care, (2) inclusion of a risk assessment to improve clinician-adolescent HIV/STI and drug use communication, (3) incorporation of culturally specific HIV/STI and drug use content, (4) incorporation of interactive aspects in the app to engage youth, and (5) perspectives on the appearance of the app. Following each theme, we describe the ways in which qualitative data were used to inform the development of the S4E app and, where applicable, how each aspect translates to the content of the original SFE intervention.

**Table 2:** Thematic analytic process.

Research question	Theme	Categories	Subcategories
“What are the facilitators and barriers to participating in an mHealth intervention in a primary care setting?”	An mHealth app is acceptable to adolescents in primary care.	Barriers to using an app	Confidentiality
		Facilitators to using an app	Informational
			Youth familiarity with technology
		Perspectives on the length and delivery of the preventive intervention	Mode of delivery
			Length
			When to deliver
			Where to deliver
“What are your thoughts about including your physician and/or parents in an mHealth preventive intervention?”	Inclusion of a risk assessment to improve clinician-adolescent HIV/STI and drug use communication	Barriers to communicating HIV/STI risk behaviors to physicians	Barriers to talking to doctors/nurses
			Methods to improve doctor/nurse communication
			Why youth do not seek help
		Facilitators to communicating HIV/STI behaviors to physicians	Why include doctors/nurses in the app
			Why it is easier to talk to doctors/nurses
			Doctors can answer questions/help you understand
“What do you believe are the most relevant drug/alcohol and sexual risk behaviors in your community that could be included in this intervention?”	Incorporation of culturally specific HIV/STI and drug use content	Specific HIV/STI to highlight	HIV/AIDS
			Gonorrhea
			Chlamydia
			Herpes
		HIV/STI risk and protective factors	Why youth are participating in risky sexual behaviors
			Why youth do not use condoms
			Relevant risky sexual behavior
			Where youth get information
			Availability of free condoms
		HIV/STI knowledge development	Birth control methods
			Signs/symptoms of STIs
			Pregnancy
			Including a doctor when talking about condoms
		Specific licit and illicit drugs to highlight	Marijuana
			Alcohol
			Prescription pills
		Drug use risk and protective factors	Relevant topics to app besides sex and drugs
			Substance use in schools
			Why youth use substances
			How to prevent substance use
		Drug use knowledge development	Long-term effects of drug use
			Drug effects
			How to stop using drugs

“What kinds of activities would you find most interesting to maintain your attention in an app?”	Incorporation of interactive aspects in the app to engage youth	Including activities to engage youth	Quiz
			Game
			Glossary/vocabulary words
			Number of activities
		Using videos to engage youth	Characteristics of the videos
			People in the videos
			Content of the videos
			Length of the videos
		Perspectives on including audio	Not including music in the app
			Including music in the app
			Type of music
“What are your opinions in terms of the kind of style the app should have? What colors do you find most engaging?”	Perspectives on the appearance of the app	Format	Different sections
			Interactive/user-friendly
			Ages to target
		Appearance of the app	Simple
			Bright colors
			Text
			Images

### Trustworthiness of Data

Trustworthiness of data is the process by which methodological rigor is employed to minimize researcher bias and ensure study findings accurately describe the participants’ perspectives [30,31]. Trustworthiness was established through credibility, transferability, dependability, and confirmability [31]. To ensure credibility, or rigor in the research process, the research team participated in prolonged engagement with participants, research reflexivity, and coanalysis. The primary author, for example, made many informal visits to the primary care clinic, including sharing meals with potential and former participants. Transferability is the extent to which study findings can be applied to other contexts. To this end, the study sample and methodological approach used to develop the app have been described in detail. Dependability is concerned with working toward explicit and repeatable study findings. This was achieved by conducting an audit trail to document research decision-making processes. Lastly, confirmability acknowledges that in qualitative research, because the researcher is the data collection instrument, there is the potential for biases. Because the data collection and app development were synergistic, this process allowed for management of subjectivity. For example, in each subsequent interview, we provided examples of design templates informed by feedback from previous interviews. This was done once participants shared their perspective and essentially served as member checks [31].

## Results

### An mHealth App is Acceptable to Adolescents in Primary Care

Participants (N=29) provided insight into a framework for the acceptability, logistical aspects, and potential barriers when using an app in primary care. Adolescents reflected on when and where to deliver the app, what platform to use, and the most appropriate length to maintain engagement.

#### Barriers and Facilitators to Using an App

Participants (N=29) were asked about the barriers and facilitators to using an app in primary care. The biggest potential barrier noted by participants was that of confidentiality. One participant expressed,

Just your overall level of comfort with sharing experiences with an electronic device is not really, to some people it might be a little bit risky, you know? Because they’re like, “I mean, who’s gonna look at this? Is this gonna be part of a statistic?” Just confidentiality.

Male, 18

Similarly, another participant expressed,

Just wondering how many people would be able to see your answers or your responses to whatever is on the app?

Female, 15

Adolescents also shared their perspectives with respect to the facilitators and acceptability of an app in primary care. Participants stated that an app was ideal because of the familiarity that youth have with technology. For example, one participant mentioned,

I think an app would be more attractive, rather than a clipboard and seven pieces of paper, you know? People our age are more gravitated towards technology and stuff like that.

Male, 18

Another adolescent expressed,

I feel like an app being used in a doctor’s office, it would be something that would be more memorable than maybe looking at a piece of paper [brochure on HIV/STI and drug use information].

Female, 16-18

#### Perspectives on the Length and Delivery of the App

Participants mentioned that to effectively deliver information, the app would have to be relatively short to maintain adolescents’ attention. One participant expressed,

I don’t think it should be extremely long, but I don’t think it should be really short. I think it should maybe be just right. You don’t want to make it where they get tired of it, but you want to have different options in that they don’t do the same one over and over again.

Female, 13-15

Similarly, another participant mentioned,

It should maybe be, like, 10 minutes, 15 minutes.

Female, 13-15

Participants also expressed their views on when and where the app would be most effectively delivered. Many adolescents mentioned the app should be delivered before seeing their doctor. One individual stated,

Deliver the app at check-in because that can also, especially if you are there for the doctor, you were to be like, say the behavior (drug use, sexual risk) to talk about today. It kind of can link in with the doctor’s appointment.

Female, 16-18

With respect to where to deliver the app, participants noted a preference for either the waiting or exam rooms while waiting for the clinician. One participant stated,

I think an exam room would be best.

Male, 16-18

Another adolescent expressed a preference to complete the app in the waiting room, as long as the mobile device had a privacy screen protector:

There are also like screen protectors that you can put on the screen so that if you look from the side, it just appears black but like if you’re looking at it straight on looks, like you can see it normal. So that might be a good idea.

Female, 17-18

#### App Development

Data provided insight into the acceptability of delivering a preventive intervention via an mHealth app in primary care. Based on adolescents’ feedback, we developed an app that aims to ensure participants’ confidentiality such that only their clinician will have access to their responses. Specifically, adolescents are assigned unique username and password information aimed at maintaining their confidentiality, including risk assessment responses. A next important step will be the production of a clinician interface to communicate and retrieve adolescent risk assessment data while maintaining compliance with Health Insurance Portability and Accountability Act standards. Furthermore, we developed an app that is relatively short in duration and can be completed during their health care visit. Specifically, the S4E app can be completed in less than 60 minutes and delivered in the clinic waiting/exam rooms.

### Inclusion of a Risk Assessment to Improve Clinician-Adolescent HIV/STI and Drug Use Communication

Participants (N=29) expressed their opinions on barriers to clinician-adolescent HIV/STI and drug use communication, and the role that an app may play in facilitating these difficult conversations.

#### Barriers to Clinician-Adolescent HIV/STI and Drug Use Communication

Participants (N=29) discussed barriers to communication and engagement in clinician-adolescent HIV/STI and drug use communication. Adolescents expressed concerns with regard to being judged by their clinician. For example, one participant stated,

Who likes to just go to somebody and say, “I’m on drugs?” That’s, like, something that you don’t really talk about a lot. So, it’s, like, why would I tell a doctor if they are going to look down on me or something, like, make you feel worse about doing it?

Female, 16-18

Another participant reflected,

Kids aren’t always honest with their doctors. They might have a reason. They might be afraid they’re going to tell their parents. They might feel like they’re gonna be judged.

Female, 16-18

#### Role of an App in Facilitating Clinician-Adolescent HIV/STI and Drug Use Communication

Participants (N=29) described the role that an app may have in facilitating clinician-adolescent HIV/STI and drug use communication. Specifically, adolescents suggested that, as part of the app, a risk assessment would help to facilitate difficult conversations, including sexual risk and drug use behaviors. For example, one participant mentioned,

A lot of people don’t say things to doctors that they’re thinking or feeling. So, if you could just do it through the app, it would be easy.

Female, 17-18

Similarly, another adolescent expressed,

I think that would be a lot better (having an assessment shared with a clinician), because some teens are kind of scared to say it face-to-face about substance use or sexual things. So, I feel like the app would be a lot better.

Female, 15

#### App Development

**Figure 1:**
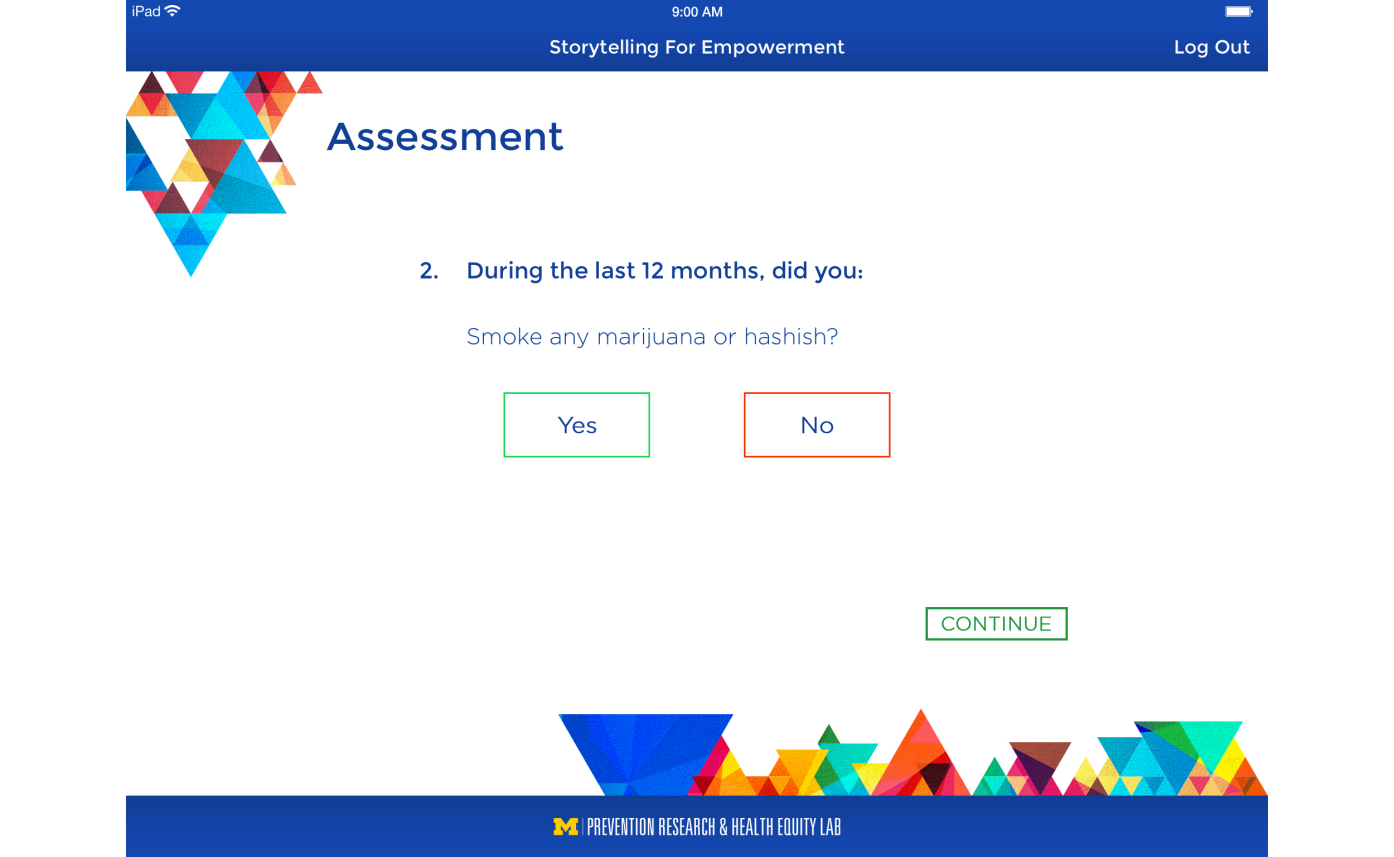
S4E risk assessment.

Data informed the development of a brief risk assessment as part of the S4E intervention. The risk assessment is completed prior to participating in the HIV/STI and Alcohol/Drug Use modules, assesses past 12-month sexual risk and alcohol and drug use behaviors, takes approximately 1 minute to complete, and will be provided to the clinician to initiate and engage the adolescent in HIV/STI and drug use communication (Figure 1).

### Incorporating Culturally Specific HIV/STI and Drug Use Content

Participants (N=29) shared the importance of culturally specific HIV/STI and drug use content that is prevalent in their community.

#### Specific HIV/STI to Highlight

Participants (N=29) suggested specific HIV/STI to highlight and areas in which they believe knowledge could be increased. For example, one adolescent stated,

I know a lot of people who are concerned about herpes, gonorrhea, chlamydia, and AIDS.

Female, 15

Similarly, another participant mentioned,

The ones (HIV/STI) that are not typically talked about, like, syphilis isn’t typically talked about. Like, chlamydia and gonorrhea is mentioned, but it’s not always talked about.

Female, 16-18

Another adolescent expressed,

With STDs, it’s not a matter of which STD to teach about, it’s a matter of how to teach about all of them. Because they are all really important.

Male, 16-18

#### HIV/STI Sexual Risk and Protective Factors

Participants (N=29) described specific HIV/STI sexual risk and protective factors that should be highlighted in the app. By far, the most frequently reported risk factor was condomless sex. One participant shared an experience of having engaged in condomless sex and stated,

I don’t know man, I wasn’t thinkin’. I wanted to see how it feels.

Male, 13-15

Another participant described peer pressure and not having condoms,

The only thing I can really think of is like peer pressure. I know like a lot of people have older friends and they might not be doing safe sex because I know I have old friends. I have friends that are having sex, so it could be peer pressure or they just don’t have a condom, and they want to have sex so bad.

Female, 13-15

Youth also shared their views with regard to HIV/STI protective factors, with a focus on self-efficacy. One participant stated,

I think it’s really important for people to understand that they are in charge of their bodies, not their doctor, not their mom, not their dad, them.

Female, 16-18

#### HIV/STI Sexual Risk Knowledge Development

Participants (N=29) reflected on the importance of the app as a vehicle to disseminate HIV/STI information, including signs and symptoms of HIV/STI, and condom use. For example, one adolescent mentioned,

I think educating people about birth control, like knowing that there is more than just condoms (is important).

Female, 16-18

Adolescents also mentioned the app should include a section on symptoms. One participant shared,

Include symptoms that you could be having and you could confuse with something more simple, like the common cold. And actually learning that the most common symptom is no symptoms at all.

Female, 17-18

#### Specific Licit and Illicit Drugs to Highlight

Adolescents (N=29) identified licit and illicit drugs that were relevant to their community. Not surprisingly, participants focused on alcohol and marijuana. One adolescent stated,

Everybody is using all different types of drugs, but marijuana is the one I see a lot.

Female, 17-18

Additionally, youth discussed prescription pills as problematic in their community. For example, one participant stated,

It’s not even sometimes specific pills. It’s just whatever you find in the cabinets of some people.

Female, 13-15

Similarly, another participant shared the following:

I think it’s a lot easier to take a pill than to smoke a blunt I guess. Because you take pills, a lot of people take pills on a daily basis. Like, it’s normal. I grew up with pills or whatever. I think it’s a lot easier to kind of deal with yourself.

Male, 16-18

#### Licit and Illicit Drug Use Risk and Protective Factors

Participants (N=29) explored potential risk and protective factors for adolescent drug use. The majority of youth identified risk and protective factors within family and peer contexts. One participant stated,

A lot of kids have problems that are going on at home and they don’t know like another way to deal with it than drugs.

Female, 18

Another adolescent noted that peer pressure has a salient influence on drug use,

I think kids our age are using drugs because, at our school, there’s a whole bunch of popular kids. And, you know, they do all that stuff. So, the kids that haven’t done it yet, they do it just to get attention from them so they can hang out with them.

Female, 13-15

With regard to protective factors, youth identified the importance of having a trusted adult in their lives. One participant mentioned,

Honestly, somebody that knows me a while that I can get to know too. Someone, like, an adult who I’ve known them my whole life. I can talk to them about personal things and all that so he can tell me what to do, give me some stuff, ideas I can do and go to.

Male, 13-15

#### Licit and Illicit Drug Use Knowledge Development

Adolescents (N=29) mentioned various myths and questions many adolescents have with respect to drug use. Participants were particularly interested in creating app content that addressed facts and effects of drugs relevant to their community. In addition, participants expressed the importance of content that aims to educate, as opposed to create fear in adolescents, as well as harm reduction approaches:

So, if you drink a lot, don’t say, “Don’t drink because of this or that.” Say, “If you are drinking, here are some steps that you can take to no longer drink or to at least not excessively drink.” Not saying, “Stop drinking completely.”

Female, 16-18

#### App Development

**Figure 2:**
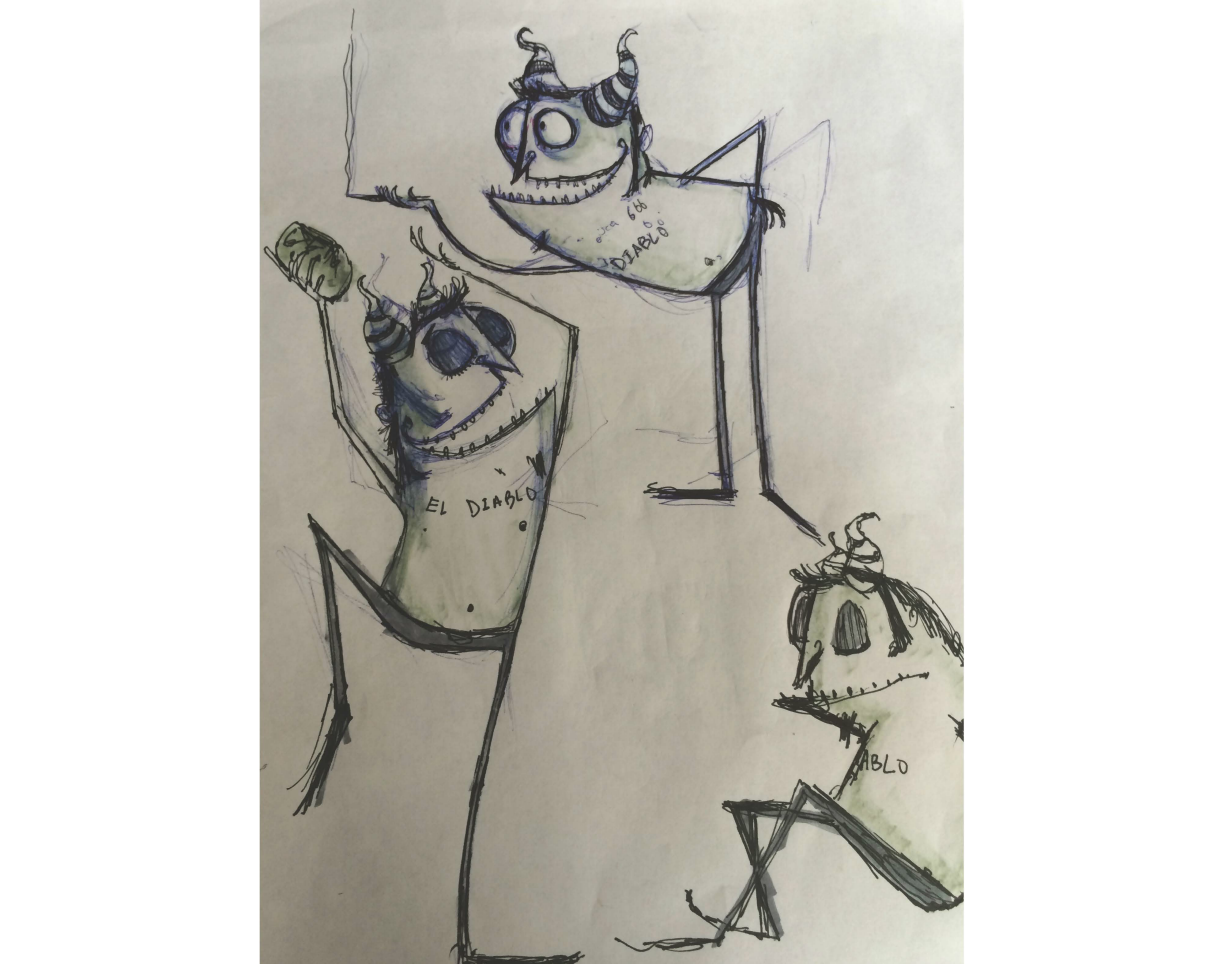
Draft model of storytelling character and video.

**Figure 3:**
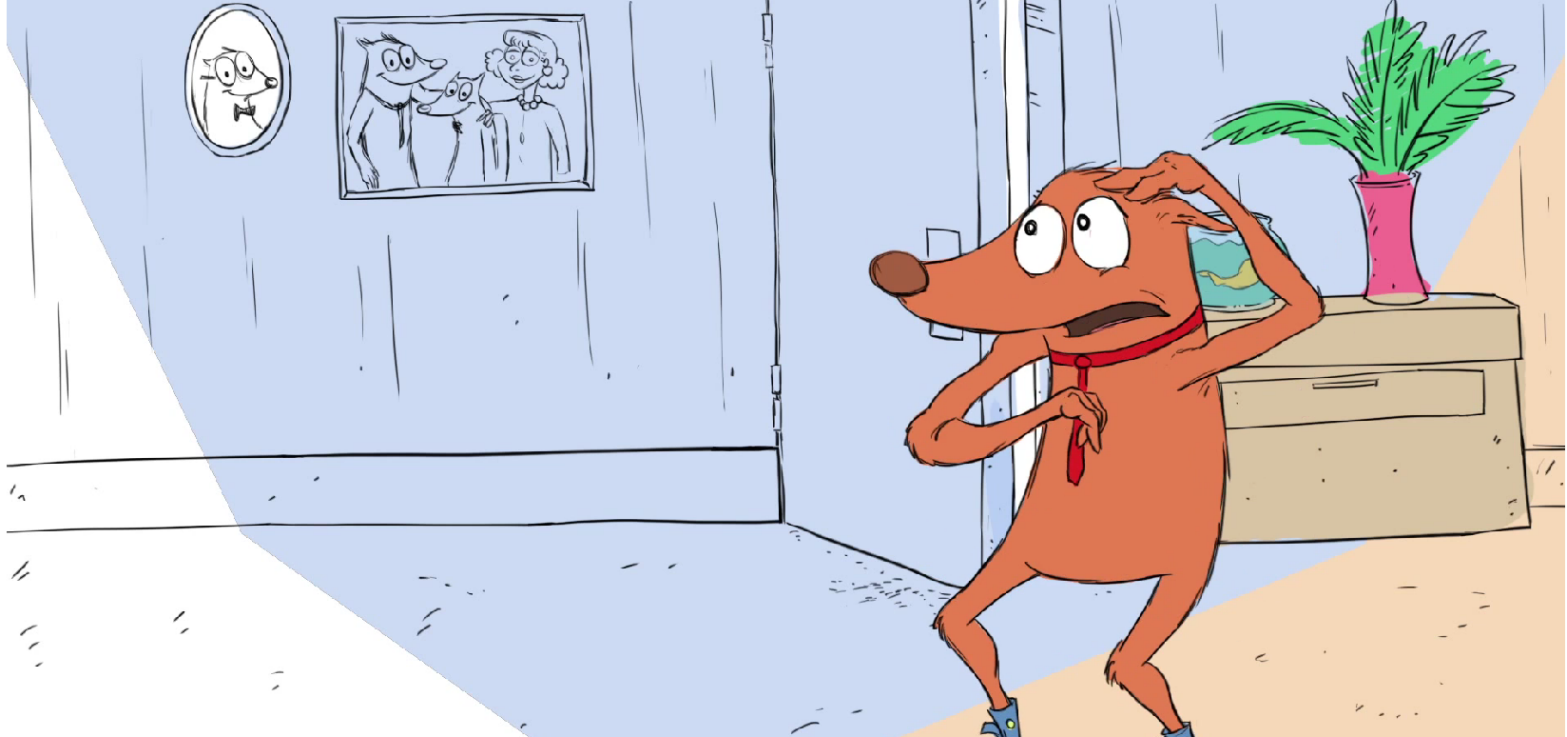
Finalized storytelling character and video.

Data were combined to develop characters and scripts for videos as part of the S4E preventive intervention. In addition, with an aim of developing culturally specific content for the targeted community, content development was informed by local HIV/STI and drug use epidemiologic data. Working closely with the Youth Leadership Council, in conjunction with the NIDA-recommended prevention principles, we developed and included videos highlighting community-specific sexual risk (eg, condomless sex) and drug use (eg, marijuana) behaviors, HIV/STI and drug use risk (eg, peer pressure) and protective (eg, self-efficacy) factors, and HIV/STI and drug use knowledge development (eg, condom demonstration, refusal skills). Draft models of characters and storylines were shown to adolescents to gather feedback, and necessary revisions were made before finalizing the stories (Figures 2 and 3). As an illustration, one video focused on 3 characters, 2 of whom engaged in unprotected sex while the third demonstrated condom use self-efficacy and refusal skills. The video highlighted HIV/STI sexual risk (ie, peer pressure) and protective (ie, self-efficacy) factors, HIV/STI sexual risk (ie, condomless sex) and protective (ie, condom use) behaviors, and unintended health consequences of HIV/STI risk behaviors (ie, acquisition and transmission of HIV/STI). Furthermore, throughout the development process, we ensured that the videos aligned with core concepts of the face-to-face version of SFE intervention (ie, knowledge-, skill-, personal-, character-, cultural-, and future-power). In this video, for example, the messaging incorporates knowledge power (ie, effects of condomless sex), skill power (ie, decision making strategies), character power (ie, positive character traits), cultural power (ie, community-specific protective factors identified in the qualitative interviews), and future power (ie, clinician-adolescent HIV/STI and drug use communication).

**Figure 4:**
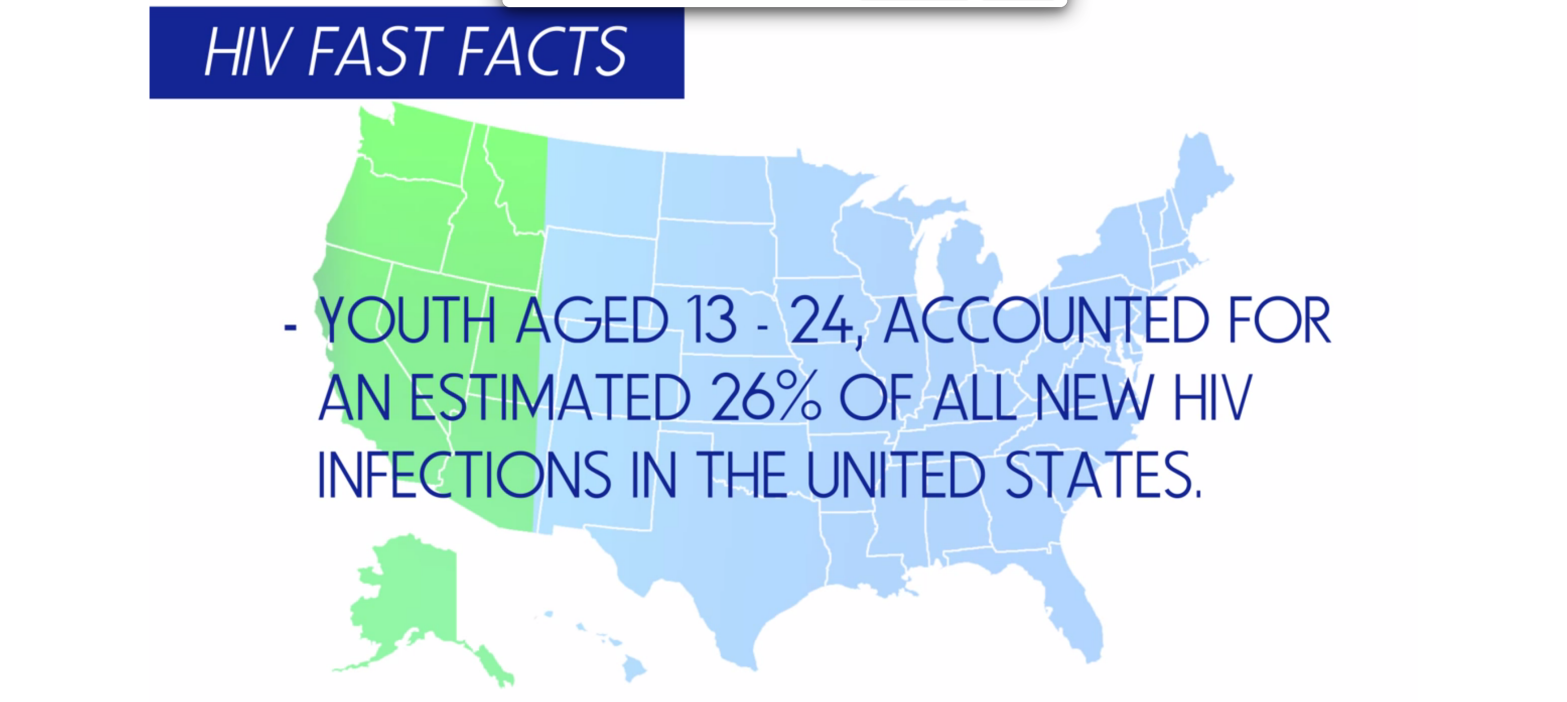
HIV/STI knowledge development.

Beyond the development of the storytelling videos, we utilized the data to develop videos aimed at increasing HIV/STI and drug use knowledge and self-efficacy. The videos focused on HIV/STI and drug use epidemiological data (eg, prevalence, etiology, effects, and symptoms) that youth identified as important to them (Figure 4).

### Incorporating Interactive Aspects in the App to Engage Youth

Participants (N=29) elaborated on interactive activities the app could include to engage youth. Familiar media sources, such as videos, audio, and creative interactive activities, were discussed as important elements to incorporate to engage youth and increase participation in the app.

#### Including Interactive Activities to Engage Youth

Youth (N=29) discussed the importance of interactive information delivery. Participants expressed that drag and drop modalities, games, and quizzes might be helpful to reinforce information provided in the app. For example, one adolescent expressed,

Just not something you would read in school. Like, long paragraphs and stuff. I think kind of more quotes and stuff would kind of make you more interested instead of paragraphs and stuff.

Female, 15

Another participant emphasized activities to keep adolescents’ interested and shared,

I don’t know if you could turn it into games, but if you could turn it into a game, do it. It will keep the interest there. I’ll play, continue to play it because it keeps me interested.

Female, 16-18

Other participants suggested using the app to evaluate the learning process:

Like, for activities, just make it more interactive. Maybe you could have a rate of test your knowledge kind of thing. You could have it where they would maybe take a pretest and they see how they get what they know, and then like at the end of looking at what you’re looking at, you could have something where it’s like a posttest.

Female, 13-15

Adolescents commented on the importance of keeping the activities youth-friendly, fun, and interactive, while also presenting important information. One participant mentioned,

I don’t think it should be serious the whole time, but it should be serious, not just all humor, so they understand it’s a topic that’s serious.

Male, 13-15

Another adolescent mentioned,

It’s a serious topic, but at the same time, it could be fun.

Male, 18

#### Using Videos to Engage Youth

Youth shared the importance of including videos to “hook” the adolescents’ interest in the app. For example, one youth stated,

I think that to make the app better, we should like put videos of people who have those problems and so we can see what people actually have to go through.

Female, 13-15

Another participant expanded,

Maybe a more literal approach to the effects and the experiences…Why you don’t want it [HIV/STI], how to get it, what you should do to prevent it…somebody that’s not afraid to open up. You need experience right there live in your face, to be like, “wow.”

Male, 18

Participants also mentioned the utility of animated videos and the ways in which these videos would help engage youth. One participant expressed,

We could try, you could have a comic strip with the characters and you could talk about drugs and stuff.

Male, 13-15

#### Perspectives on Including Audio

Youth expressed the importance of including both video and audio as part of the app processes. One participant noted,

Probably have like captions and then read to you. You can follow along with it.

Male, 13-15

Another adolescent mentioned,

I think I would learn more by hearing it.

Female, 13-15

Participants also expressed the importance of including headphones if audio were to be included as part of the app. One youth shared,

And then, because if it’s audio, then you would wanna keep it to yourself probably. Not have it out loud.

Female, 13-15

#### App Development

**Figure 5:**
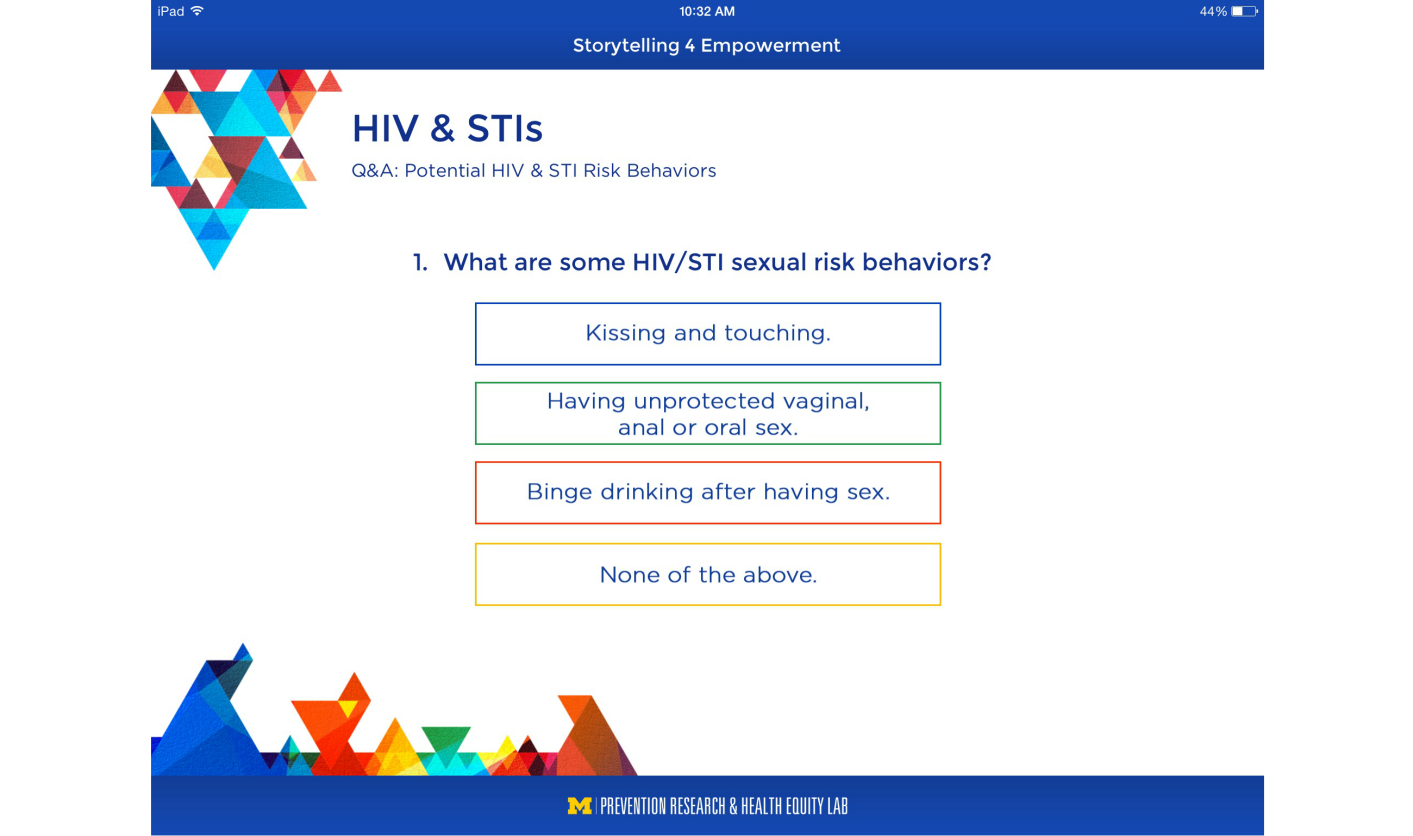
Interactive activity.

Data were used to inform the development of interactive activities, including quizzes, to maintain the adolescent’s attention and engagement in the app (Figure 5). The participants’ feedback was also used to inform the development of the storytelling videos (described earlier). Additionally, we developed an app that incorporated audio throughout the entire intervention. Therefore, in addition to the text displayed on the screen, all presented data were read aloud.

### Perspectives on the Appearance of the App

Participants (N=29) described their preferences with regard to the app appearance and format. Youth discussed the importance of an app being aesthetically engaging, including bright colors, and bold, bubble, and graffiti style text, while also being user-friendly and simple.

#### App Format

Adolescents highlighted the importance of having different sections in the app in an effort to maintain an interactive element. For example, one youth expressed,

I think it should be separate modules because it could be easier to find something.

Female, 13-15

One participant stated the importance of language when discussing how to format the app to engage youth:

Break things down in my language, and things I do. I mean, don’t say things like in my street talk, but break it down into where I can understand it would definitely be helpful.

Female, 17-18

#### Appearance of the App

Participants (N=29) expressed the need to develop an app that is aesthetically appealing to youth. Adolescents described an app that is simple with respect to user experience and bright visuals and text. One youth mentioned,

It should be colorful.

Female, 18

Similarly, another participant said,

I think bright and bold is, like, catchy.

Female, 17-18

One youth discussed the effectiveness of visuals, as compared to text-heavy sections,

If there was a lot of colors and visual, a lot of visual things that you can see in the app instead of just a lot of words with questions. Like, if there was actual images of certain things, that would draw me in.

Female, 15

#### App Development

**Figure 6:**
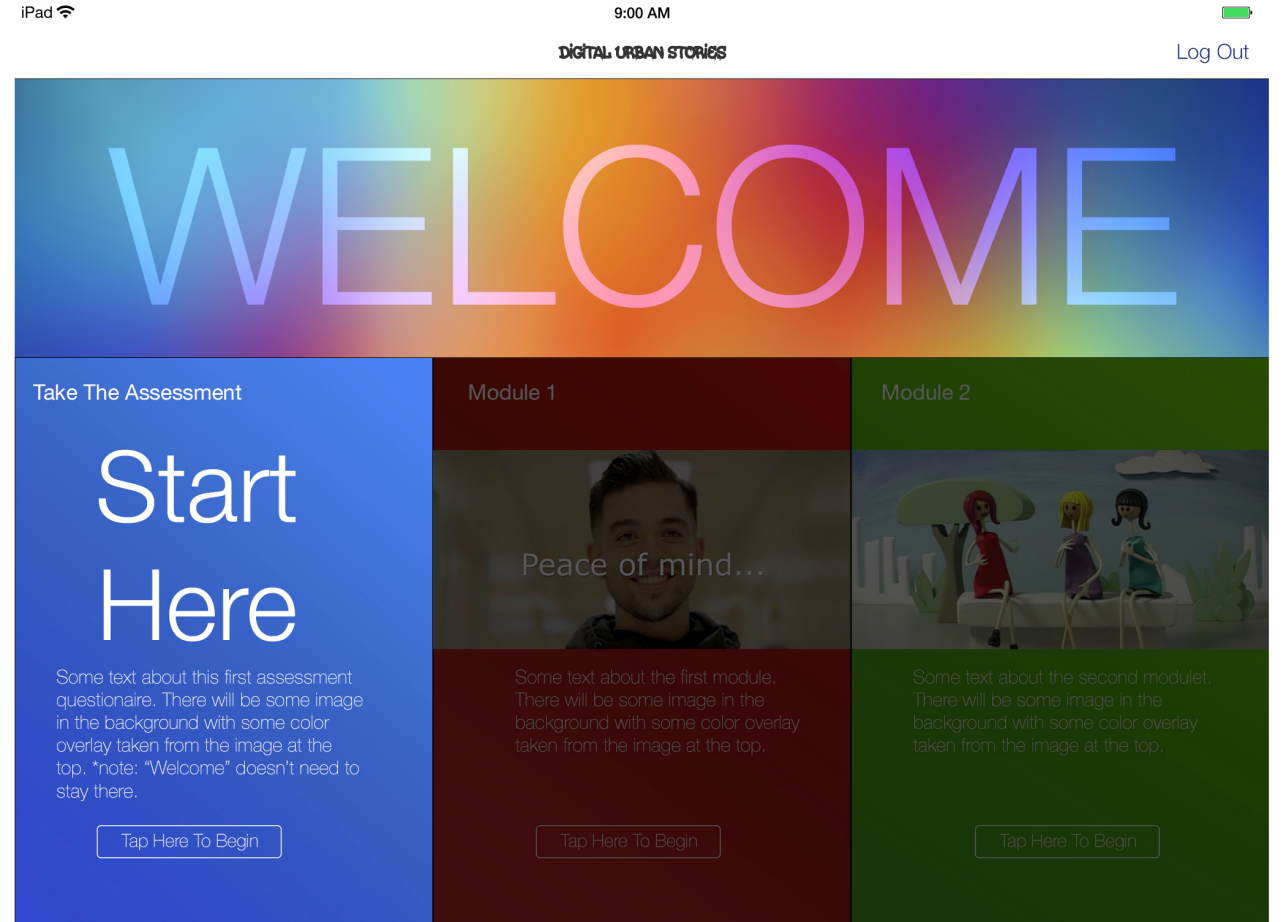
Draft model of app appearance.

**Figure 7:**
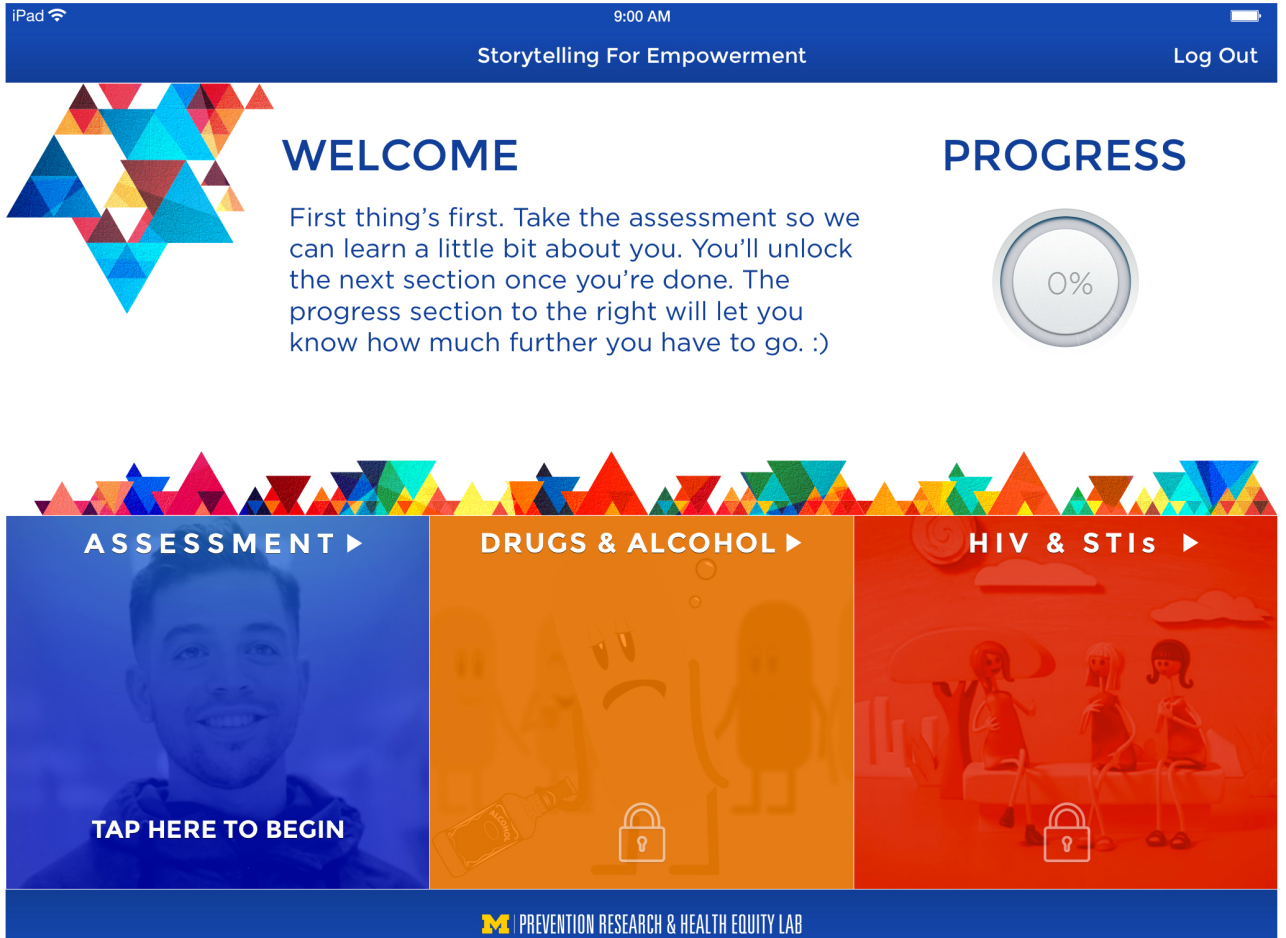
Final model of app appearance.

**Figure 8:**
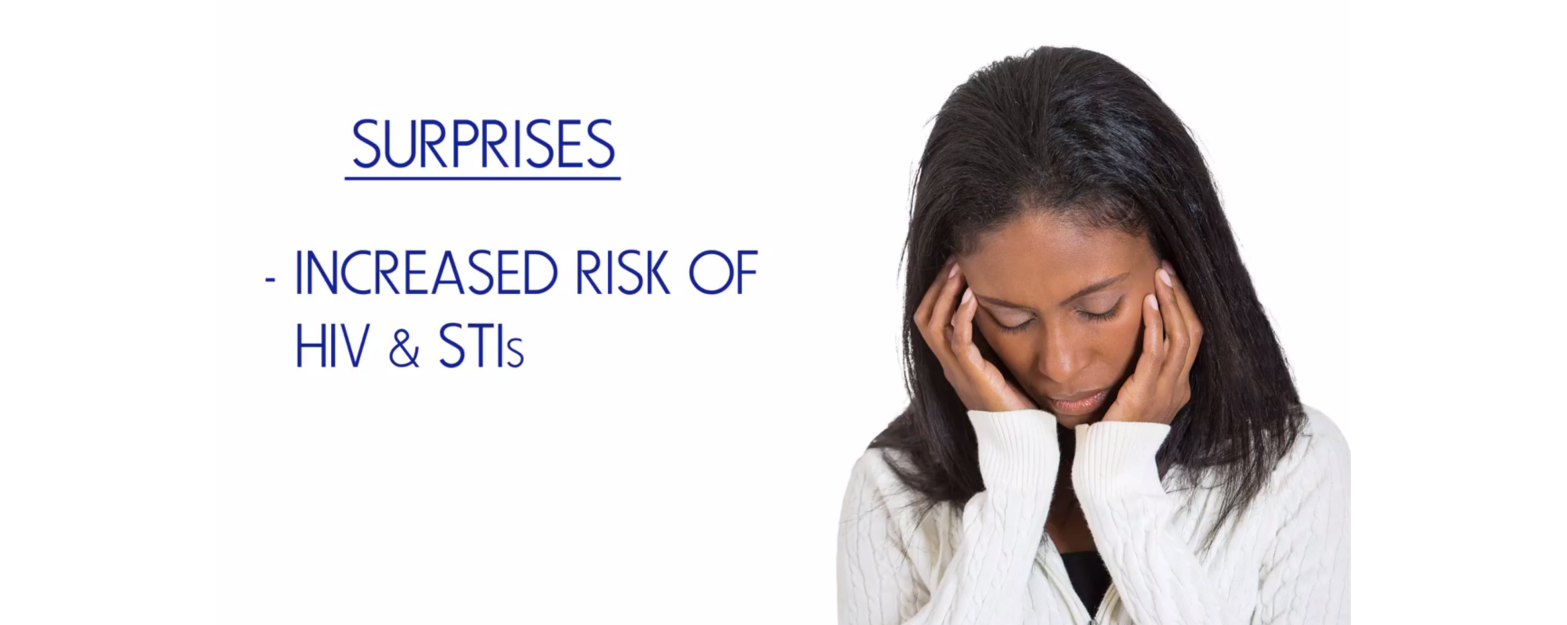
Visuals to enhance the user experience.

**Figure 9:**
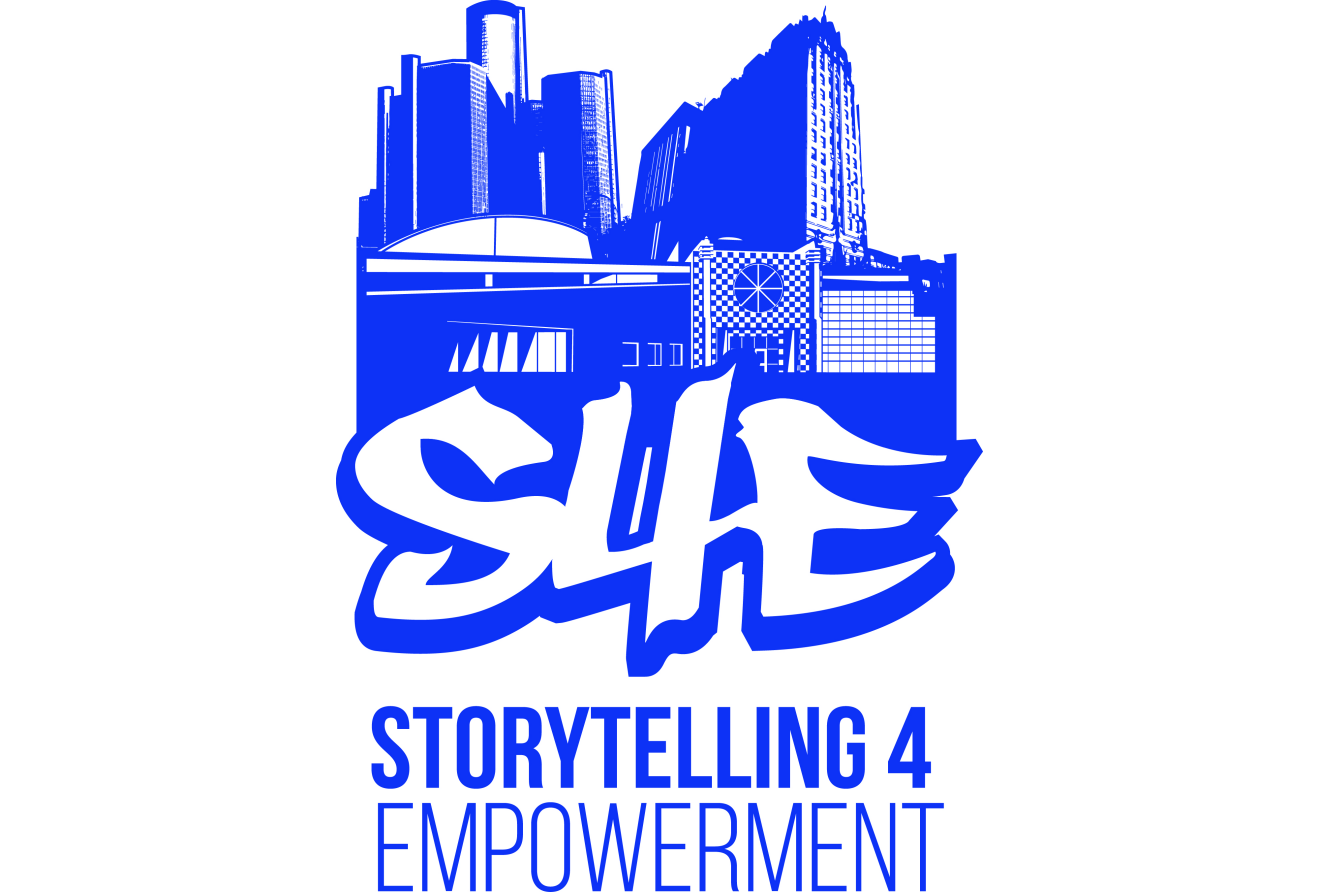
S4E logo.

Through an iterative process, we developed the framework used throughout the app. Specifically, we developed draft models of the app’s format and appearance (Figure 6), shared these models with the Youth Leadership Council and participants, and made necessary revisions based on their feedback, which ultimately led to our final model (Figure 7). In the S4E app, we minimized text and provided more visuals to enhance the user experience (Figure 8). Finally, in consultation with the Youth Leadership Council, the S4E logo was developed for branding the mHealth preventive intervention (Figure 9).

## Discussion

### Principal Findings

The purpose of this study was to develop an mHealth version of the Storytelling for Empowerment preventive intervention for primary care. Perhaps now more than ever, apps have become ubiquitous in prevention science. Yet, few apps have been developed specifically for primary care contexts. This study works to fill this gap through a community-engaged approach to gather data on the barriers and facilitators to participation in an intervention delivered via an app in primary care, as well as content, format, and processes that should be incorporated in the app design.

Findings of this study highlight important barriers and facilitators to the uptake of S4E in primary care. Specifically, adolescents expressed concern with sharing personal information, including HIV/STI risk behaviors, and the security of such information in an app platform. This finding expands on a recent report issued by the IMS Institute for Healthcare Informatics (2013) [32] by providing an adolescent consumer perspective. In that report, clinicians identified a lack of confidence in the security and privacy of patient data collected by apps as a significant concern when implementing mHealth interventions in primary care. Therefore, prior to scaling up mHealth preventive interventions, it is essential for prevention researchers to demonstrate that apps are secure and maintain the privacy of patient data. Doing so may optimize the uptake of apps by both clinicians and adolescents. With respect to facilitators, mHealth platforms have the potential to advance the delivery of HIV/STI and drug abuse preventive interventions in disproportionately affected populations, including urban youth [33]. Participants in this study expressed having access to and familiarity with technology as important facilitators for the uptake of apps in primary care. In fact, a recent report by the Pew Research Center (2015) suggested that approximately 66% of Americans are mobile phone owners, which may provide an entry point for the delivery of mHealth interventions. Furthermore, younger, ethnic and racial minority populations with relatively low income and educational attainment are more likely to be mobile phone-dependent, as compared with their counterparts [34]. Thus, mHealth approaches to HIV/STI and drug abuse prevention, including the use of mobile phones may have greater reach to those who disproportionately carry the burden of HIV/STI.

Demonstrating the utility of incorporating the principles of CBPR [35,36], agile software development [26], and NIDA-recommended core prevention principles [27] has important implications in the development of mHealth preventive interventions. Our study demonstrates that these approaches complement one another and may be helpful in developing culturally specific apps, which, in turn, have the potential to be more effectively integrated and implemented in primary care. Primary care interventions will be most efficacious/effective to the extent that they are acceptable to their target population. Our community-engaged approach aimed to give a voice to youth, who are key stakeholders in the development of the app. As such, the community provided vital information with respect to the design, content, and process of the app. These data (eg, community risk and protective factors, specific behaviors) were used to develop stories of cultural relevance to the targeted community who happened to be predominantly African American females residing in an urban area. The agile software development [26] approach allowed for a feedback loop, which was essential to ensuring cultural relevance. Specifically, because data were collected while the interface and preliminary models were built, this provided the research team with opportunities to share concept models with participants early on in the design process, and gather feedback with respect to necessary modifications. Equally important, incorporating the NIDA prevention principles ensured that the content of the app was grounded in decades of prevention research. For example, NIDA Principle 1 is concerned with enhancing protective factors and reducing risk factors [27]. Participants’ feedback informed the development of modules aimed at improving clinician-adolescent sexual risk and drug use communication (protective factor), and the potential effects of unprotected sex (risk factor).

### Future Research

This study has important research implications. For example, the development of apps has far outpaced the vetting process yet a dearth of mHealth HIV/STI and drug abuse preventive interventions have been found to be efficacious in preventing and reducing HIV/STI risk behaviors [19,37]. Therefore, an important next step is to examine the effects of S4E on HIV risk behaviors in a randomized clinical design. In addition, this study focused on adolescent perspectives, yet it may be equally important to understand clinician perspectives. Therefore, future research should examine clinician perspectives on the utility of an mHealth intervention in primary care practice. Furthermore, although we did not present the data in our results, participants’ expressed the need to include parents in prevention programs and for improved parent-adolescent HIV/STI communication. Based on participants’ feedback, it would be important to obtain parent perspectives on mHealth interventions in primary care. This research could focus on the ways in which parents could be involved, including further developing their skill set to communicate with youth about HIV/STI [6].

In light of the recent implementation of the ACA and technological advances, several important practice implications have emerged. Consistent with the American Academy of Pediatrics (AAP) recommendations of strengths-based approaches with adolescents in clinical care, mHealth modalities have the potential to facilitate clinician-adolescent HIV/STI and drug use communication, whereby clinicians can focus on adolescent strengths and protective skills aimed at preventing and reducing HIV/STI risk behaviors, and increasing HIV/STI testing [20,38]. For example, participants of this study recommended the inclusion of a risk assessment, to enable clinician-initiated conversations with adolescents with respect to their risk behaviors. Beyond the potential prevention benefits apps may have for adolescent consumers, primary care centers can also benefit. For example, mHealth modalities have the potential to relieve some of the responsibility and resource utilization in primary health care clinics, while still delivering a patient-centered intervention [39]. Furthermore, with expected increases in primary care visits as a result of the ACA, mHealth apps have the potential to engage adolescents in interventions who may not participate in prevention programs otherwise. Many barriers to integrating mHealth interventions in primary care practice remain, including the myriad of apps available on the market. As of June 2013, there were approximately 23,682 apps related to health care readily available in the Apple Store [32]. However, from a research perspective, availability may not guarantee efficacy/effectiveness. Furthermore, from a practice perspective, the number of apps may be overwhelming; it may not be feasible for clinicians to utilize multiple apps for various health risk behaviors, nor to remain informed as to which apps have undergone rigorous testing. As such, the development of apps targeting multiple health risk behaviors may prove more practical in primary care settings.

### Limitations

Findings should be interpreted in light of study limitations. First, a purposive sample was used in this study and thus is not representative of the US urban primary care adolescent population. Therefore, the transferability or generalizability of the data collected to inform the development of the app may be limited. Although the purpose of this study was to obtain adolescent perspectives on app development, a second limitation is that data on clinician perspectives were not collected. Future research should work to include the perspectives of clinicians, to develop a fuller understanding of barriers and facilitators to implementing an mHealth preventive intervention in primary care.

### Conclusions

Notwithstanding these limitations, this study represents an important step in working toward the development and testing of an mHealth version of the Storytelling for Empowerment preventive intervention for primary care. The long-term goal of research on this intervention program is to take S4E from efficacy, to effectiveness, to scaling up, in an effort to narrow and ultimately eliminate HIV/STI and drug use health disparities experienced by adolescents.

## Abbreviations

CBPR: community-based participatory research
S4E: Storytelling for Empowerment
STI: sexually transmitted infections
